# The Dynamic Use of *EGFR* Mutation Analysis in Cell-Free DNA as a Follow-Up Biomarker during Different Treatment Lines in Non-Small-Cell Lung Cancer Patients

**DOI:** 10.1155/2019/7954921

**Published:** 2019-01-23

**Authors:** Mónica Macías, Estibaliz Alegre, Gorka Alkorta-Aranburu, Ana Patiño-García, Beatriz Mateos, Maria P. Andueza, Alfonso Gúrpide, Jose M. Lopez-Picazo, Ignacio Gil-Bazo, Jose L. Perez-Gracia, Álvaro González

**Affiliations:** ^1^Service of Biochemistry, Clínica Universidad de Navarra, Av. Pio XII 36 Pamplona 31008, Spain; ^2^Navarra Institute for Health Research (IdiSNA), c/ Irunlarrea 3 31008 Pamplona, Spain; ^3^Unit of Genomics, CIMA LAB Diagnostics, University of Navarra, Av. Pio XII 55 31008 Pamplona, Spain; ^4^Department of Pediatrics, Clínica Universidad de Navarra, Av. Pio XII 36 31008 Pamplona, Spain; ^5^Department of Oncology, Clínica Universidad de Navarra, Av. Pio XII 36 31008 Pamplona, Spain; ^6^Centro de Investigación Biomédica en Red de Cáncer (CIBERONC), Av. Monforte de Lemos, 3-5. Pabellón 11. Planta 0 28029 Madrid Madrid, Spain

## Abstract

Epidermal growth factor receptor (*EGFR*) mutational testing in advanced non-small-cell lung cancer (NSCLC) is usually performed in tumor tissue, although cfDNA (cell-free DNA) could be an alternative. We evaluated *EGFR* mutations in cfDNA as a complementary tool in patients, who had already known *EGFR* mutations in tumor tissue and were treated with either EGFR-tyrosine kinase inhibitors (TKIs) or chemotherapy. We obtained plasma samples from 21 advanced NSCLC patients with known *EGFR* tumor mutations, before and during therapy with EGFR-TKIs and/or chemotherapy. cfDNA was isolated and *EGFR* mutations were analyzed with the multiple targeted cobas EGFR Mutation Test v2. *EGFR* mutations were detected at baseline in cfDNA from 57% of patients. The semiquantitative index (SQI) significantly decreased from the baseline (median = 11, IQR = 9.5-13) to the best response (median = 0, IQR = 0-0, *p* < 0.01), followed by a significant increase at progression (median = 11, IQR = 11-15, *p* < 0.01) in patients treated with either EGFR-TKIs or chemotherapy. The SQI obtained with the cobas EGFR Mutation Test v2 did not correlate with the concentration in copies/mL determined by droplet digital PCR. Resistance mutation p.T790M was observed at progression in patients with either type of treatment. In conclusion, cfDNA multiple targeted *EGFR* mutation analysis is useful for treatment monitoring in tissue of *EGFR*-positive NSCLC patients independently of the drug received.

## 1. Introduction

The most frequently observed epidermal growth factor receptor (*EGFR*) activating mutations in non-small-cell lung cancer (NSCLC) patients are exon 19 deletions and the L858R point mutation in exon 21 [1, 2]. These patients benefit from treatment with EGFR-tyrosine kinase inhibitors (TKIs) [1, 3], although development of acquired resistance is frequent [3–5]. The *EGFR* p.T790M mutation in exon 20 is found in approximately 50% of NSCLC resistant to EGFR-TKIs [6].

Assessment of *EGFR* mutations is necessary in NSCLC patients to guide the use of EGFR-TKIs [3, 7], and the gold standard is tumor tissue analysis [8]. Nevertheless, plasma cell-free DNA (cfDNA) represents an alternative to detect *EGFR* mutations [6, 9–11]. Moreover, blood can be repeatedly collected to isolate cfDNA, allowing a dynamic monitoring of the therapy efficacy and the detection of the development of resistance mutations [12–15]. cfDNA also allows the assessment of mutational heterogeneity [12, 16, 17], and the presence of *EGFR* mutant copies in cfDNA has prognostic value [12, 13]. However, a general drawback of liquid biopsy is the potential false-negative results in case of low concentration or low allelic fraction of tumor cfDNA [12, 17].

cfDNA can be analyzed using different methodologies, either genome-wide targeted analysis based on next-generation sequencing (NGS) or targeted analysis against previously known mutations using PCR assays based on digital and nondigital platforms [18]. Initially, NGS or the so-called hotspot panels could be a primary election, as occurs in mutation analysis of tissue biopsies, but this methodology is technically challenging and expensive and has prolonged turnaround times [19, 20]. Nowadays, the cobas EGFR Mutation Test v2 (Roche Molecular Systems) [21] has been approved by the FDA for the qualitative detection in plasma of exon 19 deletions or p.L858R of *EGFR*, to select patients with advanced NSCLC for treatment with EGFR-TKIs. An advantage of this real-time PCR test is that it is standardized and it can detect up to 42 *EGFR* mutations simultaneously.

Some authors recently proposed the combination of NGS versatility and ddPCR sensitivity in the follow-up of NSCLC patients with EGFR-TKI treatment, but this is a quite complex and expensive procedure [11]. Here, we investigated the utility of an intermediate strategy, the multiple targeted *EGFR* mutation analysis in plasma from NSCLC patients with already confirmed *EGFR* mutations in tissue biopsy, to achieve a more complete and personalized information oriented to treatment. We also explored if multiple targeted *EGFR* mutation analysis in plasma can be used in the follow-up of patients undergoing other treatments different from EGFR-TKIs. In addition, we have compared semiquantitative results obtained with the cobas technique with the number of copies/mL obtained by ddPCR in order to know if a correlation exists between them.

## 2. Materials and Methods

### 2.1. Patients

We selected 21 advanced NSCLC patients harboring *EGFR* mutations detected in tumor biopsy. Peripheral blood samples were collected in EDTA tubes at baseline and, when possible, at sequential time points, including the best response and progression, and during subsequent treatments (see Supplementary Figure 1) [22]. Response and tumor burden were assessed using evaluable lesions, according to RECIST v1.1 [12]. The protocol was approved by the local Ethics Committee, and all participants signed an informed consent.

### 2.2. Cell-Free DNA Extraction

For the cobas EGFR Mutation Test v2, cfDNA was isolated from 2 mL EDTA plasma using the cobas DNA Sample Preparation Kit (Roche Molecular Systems Inc., CA, USA) according to the manufacturer's instructions. For ddPCR use, cfDNA was extracted from 1 mL EDTA plasma with the MagMAX Cell-Free DNA Isolation Kit (Thermo Fisher Scientific, Madrid, Spain) according to the manufacturer's instructions.

DNA from the H1650 cell line positive for delE746-A750, from the H1975 cell line positive for p.L858R and p.T790M mutations of *EGFR*, and from peripheral blood mononuclear cells (PBMC) of a healthy volunteer was isolated with the QIAamp DNA Mini Kit (Qiagen, Venlo, Netherlands), to be used as positive and negative controls, respectively.

### 2.3. cfDNA Quantification and Fragment Size Analysis

Quantification of double-stranded cfDNA was performed using a Qubit 2.0 Fluorometer and the Qubit dsDNA HS Assay Kit (Thermo Fisher Scientific) according to the manufacturer's instructions. Concentration was reported in *μ*g/L and referred to the initial volume of plasma.

cfDNA fragment size distribution was analyzed using the DNA High Sensitivity D1000 ScreenTape (size range: 35-1,000 bp) (Agilent Technologies, Santa Clara, CA, USA) according to the manufacturer's instructions in the Agilent 2100 Bioanalyzer (Agilent Technologies). The profile of fragment sizes was analyzed using the 2100 Expert Software (Agilent Technologies).

### 2.4. EGFR Mutation Analysis in Tumor Samples

Detection of *EGFR* mutations in formalin-fixed paraffin-embedded and cytology tumor samples was performed either by real-time PCR with the therascreen EGFR RGQ PCR Kit (Qiagen) or with the NGS panel Oncomine Focus Assay (Thermo Fisher Scientific), as previously described [12].

### 2.5. EGFR Analysis in Cell-Free DNA

*EGFR* was analyzed in cfDNA for 42 different mutations with the cobas EGFR Mutation Test v2 (Roche) using the protocol provided by the manufacturer. This test kit contains both negative and positive controls, which should be run as a quality check in all assays. The PCR reactions were run on the cobas® z 480 analyzer with the cobas® 4800 software that reports automatically results as semiquantitative index (SQI) when an *EGFR* mutation is detected in ctDNA. The SQI reflects the proportion of mutated versus wild-type copies of the *EGFR* gene [23]. The SQI was derived from a dilution series containing known copy numbers of mutated *EGFR* and a fixed amount of wild-type *EGFR*, with the wild-type DNA serving as an internal control during real-time PCR [23]. The three more common *EGFR* mutations, delE746-A750, p.L858R, and p.T790M, were also analyzed by ddPCR performed in the QX200 Droplet Digital PCR system (Bio-Rad, Hercules, CA, USA) as previously described [12], with validated kits for both wild-type and mutated *EGFR* copies (Bio-Rad). Results were analyzed with the QuantaSoft Software (Bio-Rad). Fluorescence signals of blank and negative control samples were considered background and used to set up the cut-off.

### 2.6. Statistical Analysis

Data were expressed as median and interquartile range (IQR). Nonparametric statistical analysis was performed using GraphPad Prism version 6.07 (La Jolla, CA, USA). The Wilcoxon signed-rank test was used to evaluate the evolution of the cfDNA SQI in each patient, and the Mann-Whitney *U* test was used to compare copy levels between different patient groups. Correlation analysis between SQI results from the cobas system and copies/mL from ddPCR was performed with the Spearman test. A two-tailed *p* value of <0.05 was considered to be statistically significant.

## 3. Results

### 3.1. Clinical Characteristics of the Patients

In this retrospective study, we included 21 NSCLC patients (13 males, 58 ± 12 years old; 8 females, 63 ± 11 years old) presenting *EGFR* mutations in their tumor samples. All patients were stages III-IV, 19 had adenocarcinoma and 2 had squamous carcinoma, and 52% were never smokers. Patients were treated with either EGFR-TKIs (20 treatments) or chemotherapy (10 treatments) (see Supplementary Table 1).

### 3.2. Characterization of cfDNA: Quantification and Fragment Size

**Figure 1:**
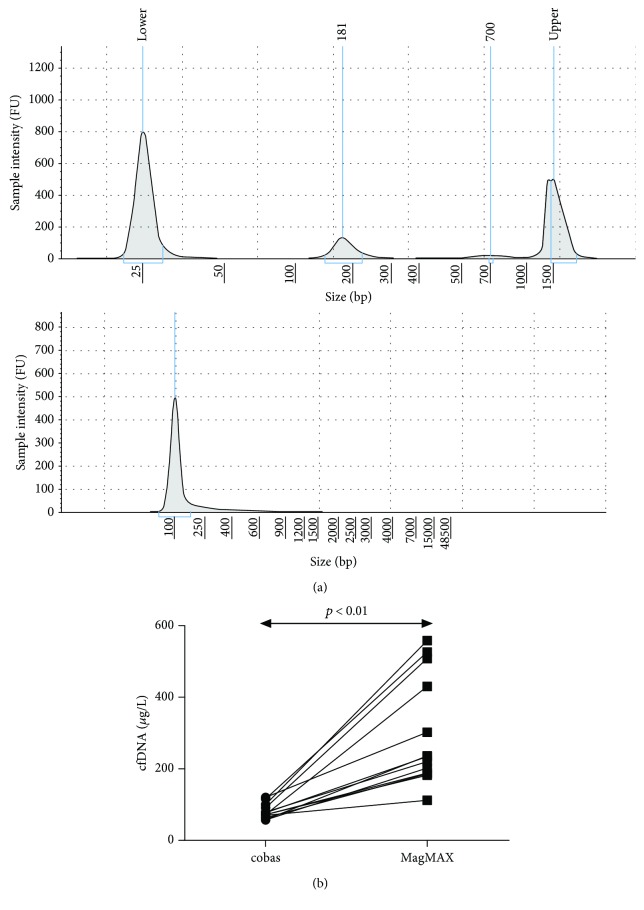
(a) Electropherogram of cfDNA samples using the High Sensitivity D1000 ScreenTape® (up) and Genomic DNA ScreenTape® (down). The upper 181 bp peak corresponds to the predominant cfDNA. (b) Comparison of the cfDNA concentrations obtained using the Roche and Thermo Fisher methods for cfDNA extraction from paired plasma samples.

The cobas EGFR Mutation Test v2 requires cfDNA obtained with the cobas DNA Sample Preparation Kit, so initially we analyzed DNA extracted using this method. The cfDNA size was 175 ± 9 bp, with a complete absence of genomic DNA (Figure 1(a)). Using 13 plasma samples, we compared its efficiency of extraction with that of MagMAX Cell-Free DNA Isolation Kit, concluding that MagMAX yielded significantly more cfDNA (*p* < 0.01) (Figure 1(b)), also free of genomic DNA (data not shown).

The median cfDNA concentration in baseline samples was 100 *μ*g/L (IQR = 61-158 *μ*g/L). The total cfDNA concentration did not change significantly during treatment: at the best response, it was 103 *μ*g/L (IQR = 79-229 *μ*g/L) and at progression, it was 84 *μ*g/L (IQR = 72-124 *μ*g/L). There was no correlation between cfDNA concentration and tumor burden (data not shown).

### 3.3. EGFR Analysis in cfDNA with the cobas EGFR Mutation Test v2

We were able to detect *EGFR* mutations in the cfDNA of 12 out of 21 patients at baseline (57%). There were no statistically significant differences in cfDNA levels between those with (median cfDNA = 120 *μ*g/L, IQR = 89-270 *μ*g/L) or without (median cfDNA = 96 *μ*g/L, IQR = 77-170 *μ*g/L) detectable *EGFR* mutations. In addition, considering the group of patients with detectable activating *EGFR* mutations in cfDNA, a complete concordance with the mutation pattern in tissue was found (12/12).

**Figure 2:**
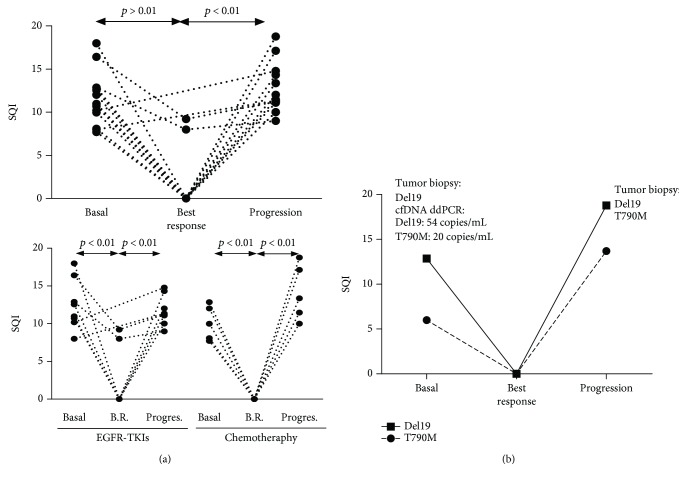
(a) Upper: longitudinal study of *EGFR* mutational levels (SQI) at baseline (*n* = 14), during treatment at the moment of the best response (*n* = 12), and at progression (*n* = 13) of the disease. Lower: longitudinal study of *EGFR* mutational levels (SQI) considering therapy with either EGFR-TKIs (Basal, *n* = 9; B.R., *n* = 7; and Progres., *n* = 8) or chemotherapy (Basal, *n* = 5; B.R., *n* = 5; and Progress, *n* = 5). (b) Evolution of the *EGFR* mutational levels (SQI) in a patient during chemotherapy (docetaxel) treatment.

In baseline samples from patients bearing activating *EGFR* mutations, the SQI decreased significantly between the baseline (median = 11, IQR = 9.5-13) and the best response (median = 0, IQR = 0-0, *p* < 0.01) (Figure 2(a)). At progression, the SQI increased significantly again (median = 11, IQR = 11-15, *p* < 0.01). Similar results were observed when patients were divided according to the type of treatment.

We also detected the p.T790M mutation in 2/21 patients at baseline (9%), and, interestingly, in one of them, the mutation had not been previously detected in the tumor biopsy. In this case, the interval between tumor biopsy and liquid biopsy was 31 months. The presence of this mutation in cfDNA was confirmed by ddPCR (Figure 2(b)). During treatment with chemotherapy, a repeated tissue biopsy 14 months afterwards confirmed the presence of delE746-A750 and p.T790M mutations, overlapping with an increase in the SQI in cfDNA found in its plasma-matched sample.

**Figure 3:**
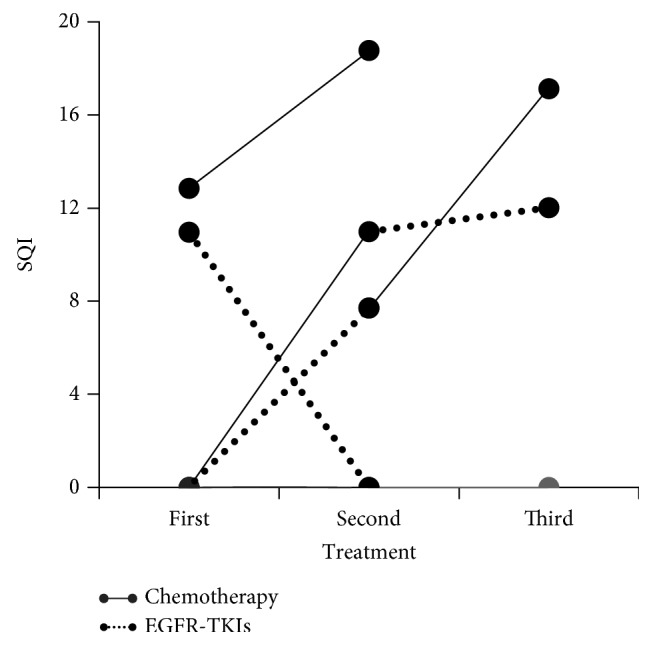
Longitudinal study of *EGFR* mutational levels (SQI) during successive treatments.

During treatment, the p.T790M mutation appeared in 4/19 (21%) patients that did not carry this mutation at baseline, either during chemotherapy (3/19) or therapy with EGFR-TKIs (1/19). p.T790M mutation remained positive in the two patients that were already positive at baseline. Regarding exon 19 deletions, they appeared at progression in 3/9 (33%) of the patients that were negative for this mutation at baseline. In addition, in 2 out of 7 patients who received a further line of treatment, exon 19 deletions were detected at the second time point but not at baseline (Figure 3).

To test for a potential correlation between the SQI reported by the cobas EGFR Mutation Test v2 and the number of mutant copies/mL reported by ddPCR, the results obtained in those samples that were positive for both methods were compared, but no significant correlation was found (*r* = 0.143).

## 4. Discussion

The implementation of molecular analytical tests with verified accuracy is important to exploit the possibilities of personalized medicine. The cobas EGFR Mutation Test v2 is a standardized methodology showing good performance compared with other techniques [17, 21]. However, the extraction methods can substantially influence the quality and quantity of the cfDNA extracted [24, 25]. The recommended method for cfDNA extraction for the cobas EGFR Mutation Test v2 recovered mainly mononucleosomic cfDNA [26], but the efficiency was lower than that observed with the MagMAX methodology. This is relevant since it could yield false-negative results when the cfDNA concentration is low [11]. In this sense, it could be interesting that the cobas EGFR Mutation Test v2 was opened to be used with other extraction kits with better performance.

Tumor tissue analysis is the gold standard to analyze *EGFR* mutations in NSCLC [8], and blood cannot be used as a surrogate source of analysis, as its sensitivity is not enough [12, 13] and depends on the platform used and the mutations analyzed [17, 27]. For example, in a previous comparison of plasma p.T790M detection using four different platforms, Thress et al. showed that ddPCR sensitivity was only of 71% with a specificity of 83%, even though it outperformed those of cobas, therascreen, and BEAMing [17]. Nevertheless, *EGFR* cfDNA analysis could be a complementary approach if the biopsy has not been recently obtained or is no longer available, thus allowing the mutational information update, as we observed here. This is especially relevant when using different lines of treatment, since previous treatments can select tumor clones and change the mutational landscape [15, 18].

We and others have shown the clinical utility of mutated *EGFR* analysis in cfDNA obtained from NSCLC patients during EGFR-TKI treatment [12, 13]. Once the mutation is identified in tissue, liquid biopsy can be also useful to monitor the treatment, since the mutation load decreases when the patient responds to therapy and increases again at progression. The cobas EGFR Mutation Test v2 only reports the semiquantitative value SQI while ddPCR can report an absolute quantification of mutant copies/mL. Although Mok et al. have used the cobas EGFR Mutation Test v2 data as an equivalent to the number of copies/mL [28], we have not observed this correspondence and, in our hands, both values are not interchangeable. Nevertheless, the effectiveness of the treatment was reflected in a decrease in the SQI of activating mutations similar to that observed in the concentration of circulating *EGFR* mutant copies as previously observed by ddPCR [12, 13]. In addition, the use of a commercially available and approved multiple targeted test for *EGFR* mutations in cfDNA allows a rapid turnaround time of one day [29]. Although we have performed this study using one FDA-approved platform, probably similar results would be obtained using other methods for multiple *EGFR* mutation test analysis.

Previous studies have assessed the utility of *EGFR* mutation analysis in patients undergoing EGFR-TKI therapy, but here we report several cases of patients treated with chemotherapy that were effectively monitored using cfDNA. This observation is relevant because *EGFR*-positive patients do not only receive EGFR-TKIs but also other treatment regimens based on chemotherapy [5]. Consequently, we can consider activating mutations in cfDNA as a potential biomarker to monitor these patients. Moreover, we have shown the appearance of the p.T790M mutation in patients receiving not only EGFR-TKIs but also chemotherapy. Other authors have shown that the p.T790M resistance mutation is only found in the cfDNA of erlotinib-treated NSCLC patients if they have an activating *EGFR* mutation before treatment [30]. Also, in NSCLC patients with activating *EGFR* mutation, longitudinal tumor rebiopsy has shown the appearance of p.T709M mutation during chemotherapy treatment [31]. A drawback with these targeted methods is that they do not check for the presence of other potential genetic causes of resistance, such as *MET* amplification [5].

In summary, here we show that multiple targeted *EGFR* mutation analysis in cfDNA can be helpful in those patients with already detected *EGFR* mutations in tissue. cfDNA analysis helps to evaluate mutational status, especially if the tissue biopsy is not recent. The availability of baseline and sequential samples also allows the monitoring of the efficacy of therapy and the detection of resistance mutations.

## Data Availability

The data from cobas and ddPCR analysis and cfDNA isolation results used to support the findings of this study are available from the corresponding author upon request. Patient characteristics are detailed in Supplementary Materials.

## Abbreviations

*EGFR*: Epidermal growth factor receptor
NSCLC: Non-small-cell lung cancer
cfDNA: Cell-free DNA
TKIs: Tyrosine kinase inhibitors
SQI: Semiquantitative index
ddPCR: Droplet digital polymerase chain reaction
NGS: Next-generation sequencing
RECIST: Response evaluation criteria in solid tumors
IQR: Interquartile range.

## References

[1] Tsao M. S., Sakurada A., Cutz J. C. (2005). Erlotinib in lung cancer - molecular and clinical predictors of outcome. *The New England Journal of Medicine*.

[2] Sheikine Y., Rangachari D., McDonald D. C. (2016). EGFR testing in advanced non-small-cell lung cancer, a mini-review. *Clinical Lung Cancer*.

[3] Novello S., Barlesi F., Califano R. (2016). Metastatic non-small-cell lung cancer: ESMO Clinical Practice Guidelines for diagnosis, treatment and follow-up. *Annals of Oncology*.

[4] Jänne P. A., Yang J. C.-H., Kim D.-W. (2015). AZD9291 in EGFR inhibitor-resistant non-small-cell lung cancer. *New England Journal of Medicine*.

[5] Lim S. M., Syn N. L., Cho B. C., Soo R. A. (2018). Acquired resistance to EGFR targeted therapy in non-small cell lung cancer: mechanisms and therapeutic strategies. *Cancer Treatment Reviews*.

[6] Rosell R., Karachaliou N. (2016). Implications of blood-based T790M genotyping and beyond in epidermal growth factor receptor-mutant non-small-cell lung cancer. *Journal of Clinical Oncology*.

[7] Keedy V. L., Temin S., Somerfield M. R. (2011). American Society of Clinical Oncology provisional clinical opinion: epidermal growth factor receptor (EGFR) mutation testing for patients with advanced non-small-cell lung cancer considering first-line EGFR tyrosine kinase inhibitor therapy. *Journal of Clinical Oncology*.

[8] Pirker R., Herth F. J., Kerr K. M. (2010). Consensus for EGFR mutation testing in non-small cell lung cancer: results from a European workshop. *Journal of Thoracic Oncology*.

[9] Oxnard G. R., Thress K. S., Alden R. S. (2016). Association between plasma genotyping and outcomes of treatment with osimertinib (AZD 9291) in advanced non-small-cell lung cancer. *Journal of Clinical Oncology*.

[10] Sorensen B. S., Wu L., Wei W. (2014). Monitoring of epidermal growth factor receptor tyrosine kinase inhibitor-sensitizing and resistance mutations in the plasma DNA of patients with advanced non-small cell lung cancer during treatment with erlotinib. *Cancer*.

[11] Iwama E., Sakai K., Azuma K. (2017). Monitoring of somatic mutations in circulating cell-free DNA by digital PCR and next-generation sequencing during afatinib treatment in patients with lung adenocarcinoma positive for EGFR activating mutations. *Annals of Oncology*.

[12] Alegre E., Fusco J. P., Restituto P. (2016). Total and mutated EGFR quantification in cell-free DNA from non-small cell lung cancer patients detects tumor heterogeneity and presents prognostic value. *Tumour Biology*.

[13] Oxnard G. R., Paweletz C. P., Kuang Y. (2014). Noninvasive detection of response and resistance in EGFR-mutant lung cancer using quantitative next-generation genotyping of cell-free plasma DNA. *Clinical Cancer Research*.

[14] Alegre E., Martínez D., Macías M., González Á. (2016). Are we ready to introduce T790M plasma analysis in the follow up of patients with NSCLC under treatment with EGFR-TKI?. *Annals of Translational Medicine*.

[15] Wan J. C. M., Massie C., Garcia-Corbacho J. (2017). Liquid biopsies come of age: towards implementation of circulating tumour DNA. *Nature Reviews. Cancer*.

[16] Jakobsen J. N., Santoni-Rugiu E., Ravn J., Sorensen J. B. (2013). Intratumour variation of biomarker expression by immunohistochemistry in resectable non-small cell lung cancer. *European Journal of Cancer*.

[17] Thress K. S., Brant R., Carr T. H. (2015). EGFR mutation detection in ctDNA from NSCLC patient plasma: a cross-platform comparison of leading technologies to support the clinical development of AZD9291. *Lung Cancer*.

[18] Macías M., Alegre E., Díaz-Lagares A. (2018). Liquid biopsy: from basic research to clinical practice. *Advances in Clinical Chemistry*.

[19] Siravegna G., Marsoni S., Siena S., Bardelli A. (2017). Integrating liquid biopsies into the management of cancer. *Nature Reviews Clinical Oncology*.

[20] Perakis S., Auer M., Belic J., Heitzer E. (2017). Advances in circulating tumor DNA analysis. *Advances in Clinical Chemistry*.

[21] Malapelle U., Sirera R., Jantus-Lewintre E. (2017). Profile of the Roche cobas® EGFR mutation test v2 for non-small cell lung cancer. *Expert Review of Molecular Diagnostics*.

[22] Perez-Gracia J. L., Sanmamed M. F., Bosch A. (2017). Strategies to design clinical studies to identify predictive biomarkers in cancer research. *Cancer Treatment Reviews*.

[23] Marchetti A., Palma J. F., Felicioni L. (2015). Early prediction of response to tyrosine kinase inhibitors by quantification of EGFR mutations in plasma of NSCLC patients. *Journal of Thoracic Oncology*.

[24] Garcia J., Dusserre E., Cheynet V. (2017). Evaluation of pre-analytical conditions and comparison of the performance of several digital PCR assays for the detection of major EGFR mutations in circulating DNA from non-small cell lung cancers: the CIRCAN_0 study. *Oncotarget*.

[25] Malentacchi F., Pizzamiglio S., Verderio P. (2015). Influence of storage conditions and extraction methods on the quantity and quality of circulating cell-free DNA (ccfDNA): the SPIDIA-DNAplas External Quality Assessment experience. *Clinical Chemistry and Laboratory Medicine*.

[26] Heitzer E., Ulz P., Geigl J. B. (2014). Circulating tumor DNA as a liquid biopsy for cancer. *Clinical Chemistry*.

[27] Karlovich C., Goldman J. W., Sun J. M. (2016). Assessment of EGFR mutation status in matched plasma and tumor tissue of NSCLC patients from a phase I study of rociletinib (CO-1686). *Clinical Cancer Research*.

[28] Mok T., Wu Y. L., Lee J. S. (2015). Detection and dynamic changes of EGFR mutations from circulating tumor DNA as a predictor of survival outcomes in NSCLC patients treated with first-line intercalated erlotinib and chemotherapy. *Clinical Cancer Research*.

[29] Rosell R., Karachaliou N. (2016). Using ctDNA to track EGFR and KRAS mutations in advanced-stage disease. *Nature Reviews Clinical Oncology*.

[30] Demuth C., Madsen A. T., Weber B., Wu L., Meldgaard P., Sorensen B. S. (2018). The T790M resistance mutation in EGFR is only found in cfDNA from erlotinib-treated NSCLC patients that harbored an activating EGFR mutation before treatment. *BMC Cancer*.

[31] Kuiper J. L., Heideman D. A. M., Thunnissen E. (2014). Incidence of T790M mutation in (sequential) rebiopsies in EGFR-mutated NSCLC-patients. *Lung Cancer*.

