# Supplementary material for: The Dynamic Use of EGFR Mutation Analysis in Cell-Free DNA as a Follow-Up Biomarker during Different Treatment Lines in Non-Small-Cell Lung Cancer Patients
Source: Dis Markers. 2019 Jan 23;2019:7954921. doi: 10.1155/2019/7954921 (PMC6364099; doi:10.1155/2019/7954921)
Supplement: Supplementary Materials — Supplementary figure and table are provided. [file 7954921.f1.zip › mat.7954921.v2.docx]

**Supplementary Table 1: Demographic characteristics of patients with advanced NSCLC and mutation detected in basal tissue biopsy.** Ex= Former smoker; M= Male; F= Female

| **Patient** | **Smoke** | **Age** | **Gender** | **Stage** | **Histopathological classification** | **Therapy** | | **Mutation detected in basal tissue biopsy** | **Time elapsed between tissue an blood biopsy** |
| --- | --- | --- | --- | --- | --- | --- | --- | --- | --- |
| 1 | No | 69 | F | IV | Adenocarcinoma | 1º line | TKI (Erlotinib) | L858R | Same day |
|  |  |  |  |  |  | 2º line | TKI (Afatinib) |  |  |
|  |  |  |  |  |  | 3º line | Chemotherapy (Carboplatin/Pemetrexed) |  |  |
| 2 | Ex | 54 | F | IV | Adenosquamous carcinoma | 1º line | TKI (Afatinib) | L858R T790M | 12 months |
| 3 | No | 75 | M | IV | Adenocarcinoma | 1º line | TKI (Afatinib) | del E746-A750 | 16 months |
|  |  |  |  |  |  | 2º line | Chemotherapy (Carboplatin) |  |  |
| 4 | No | 69 | F | IV | Adenocarcinoma | 1º line | TKI (Erlotinib) | delL747-P753 insP | 1 month |
| 5 | No | 74 | F | IV | Adenocarcinoma | 1º line | Chemotherapy (Cisplatin/Pemetrexed) | del E746-A750 | 32 months |
| 6 | Ex | 64 | M | IV | Adenocarcinoma | 1º line | Chemotherapy (Docetaxel) | del E746-A750 | 31 months |
|  |  |  |  |  |  | 2º line | Inmunotherapy (Nivolumab) |  |  |
| 7 | Yes | 53 | M | IIIA | Adenocarcinoma | 1º line | Chemotherapy (Cisplatin/Pemetrexed) | delL747-P753 insP | 1 month |
|  |  |  |  |  |  | 2º line | TKI (Erlotinib) |  |  |
|  |  |  |  |  |  | 3º line | Chemotherapy (Carboplatin/Pemetrexed) |  |  |
| 8 | Ex | 52 | F | IV | Squamous carcinoma | 1º line | TKI (Erlotinib) | L858R | 3 months |
| 9 | Ex | 54 | M | IV | Adenocarcinoma | 1º line | TKI (Erlotinib) | del E746-A750 | 6 days |
| 10 | No | 76 | F | IIIA | Adenocarcinoma | 1º line | Chemotherapy (Cisplatin/Pemetrexed) | del E746-A750 | 7 days |
|  |  |  |  |  |  | 2º line | TKI (Afatinib) |  |  |
| 11 | Ex | 45 | F | IV | Adenocarcinoma | 1º line | TKI (Erlotinib) | Del19 | Same day |
|  |  |  |  |  |  | 2º line | TKI (Erlotinib) + anti-MET (Emibetuzumab) |  |  |
| 12 | Ex | 64 | F | IV | Adenocarcinoma | 1º line | TKI (Gefitinib) | Del19 | 7 days |
| 13 | Ex | 49 | M | IV | Adenocarcinoma | 1º line | TKI (Erlotinib) | Del19 | Same day |
| 14 | No | 78 | F | IV | Adenocarcinoma | 1º line | TKI (Erlotinib) | del19 | 18 days |
| 15 | No | 41 | M | IV | Adenocarcinoma | 1º line | TKI (Erlotinib) | del E746-A750 | 3 days |
| 16 | No | 61 | F | IIIA | Adenocarcinoma | 1º line | Chemotherapy (Carboplatin/Pemetrexed) | L858R | 2 months |
|  |  |  |  |  |  | 2º line | TKI (Erlotinib) |  |  |
| 17 | No | 70 | M | IV | Adenocarcinoma | 1º line | TKI (Erlotinib) | Del19 | 12 days |
| 18 | Ex | 60 | F | IV | Adenocarcinoma | 1º line | Chemotherapy (Docetaxel/Nindenatinib) | Del19 | 2 months |
| 19 | No | 75 | F | IV | Adenocarcinoma | 1º line | TKI (Gefinitib) | Del19 | 7 days |
| 20 | Ex | 50 | F | IV | Adenocarcinoma | 1º line | TKI (Erlotinib) | Del19 | 14 days |
| 21 | No | 53 | M | IV | Adenocarcinoma | 1º line | TKI (Gefitinib) | Del19 | 2 days |
