# Supplementary material for: The Dynamic Use of EGFR Mutation Analysis in Cell-Free DNA as a Follow-Up Biomarker during Different Treatment Lines in Non-Small-Cell Lung Cancer Patients
Source: Dis Markers. 2019 Jan 23;2019:7954921. doi: 10.1155/2019/7954921 (PMC6364099; doi:10.1155/2019/7954921)
Supplement: Supplementary Materials — Supplementary figure and table are provided. [file 7954921.f1.zip › Supplementary Figure.pptx]

## Slide 1
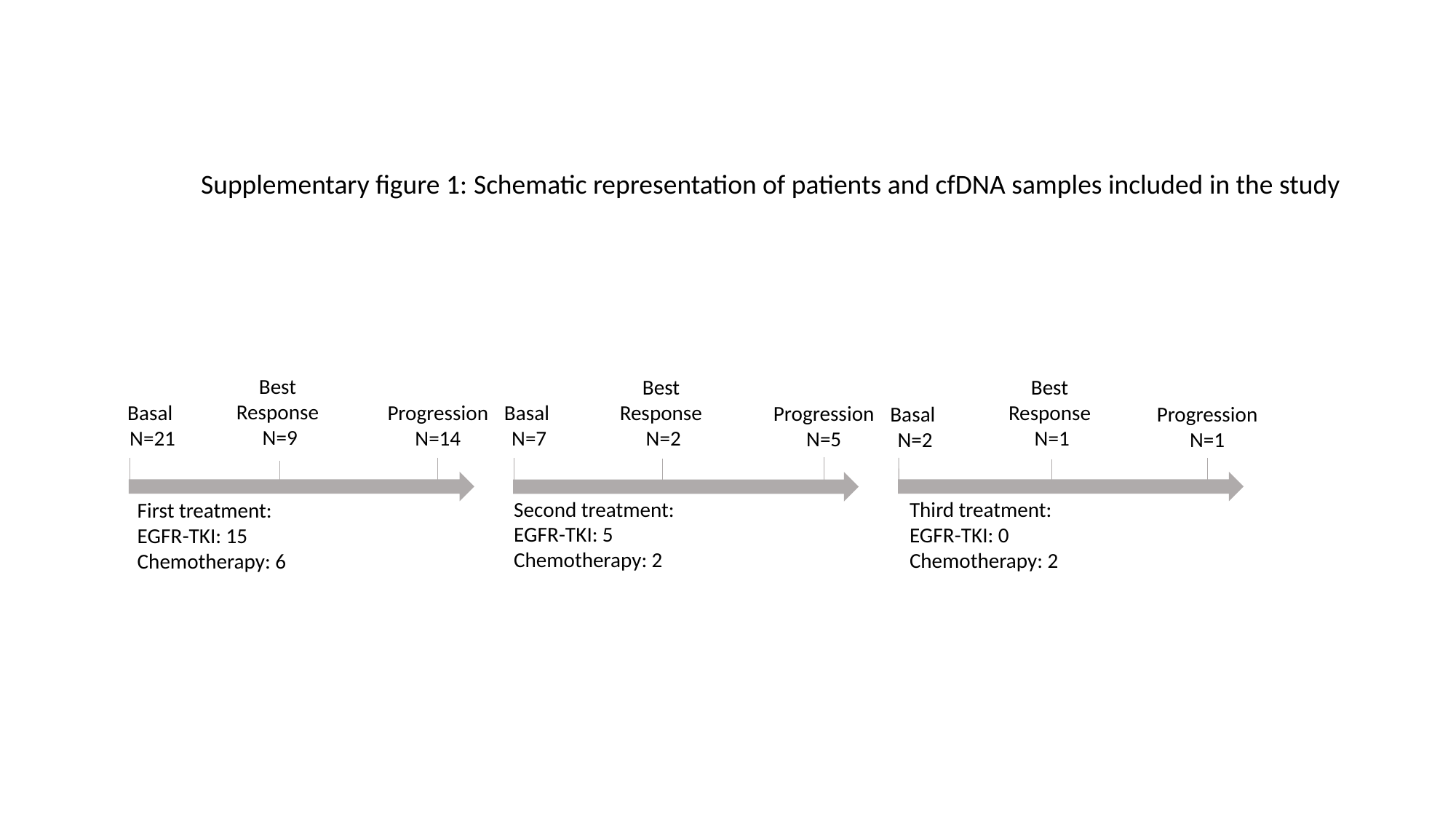

Supplementary figure 1: Schematic representation of patients and cfDNA samples included in the study
Best
Response
N=9
Basal
N=21
Progression
N=14
First treatment:
EGFR-TKI: 15
Chemotherapy: 6
Best
Response
N=2
Basal
N=7
Progression
N=5
Second treatment:
EGFR-TKI: 5
Chemotherapy: 2
Best
Response
N=1
Basal
N=2
Progression
N=1
Third treatment:
EGFR-TKI: 0
Chemotherapy: 2
